# Relative age effect at Concacaf championships: Influence of sex, age, nationality, playing position, and playing status

**DOI:** 10.1371/journal.pone.0321245

**Published:** 2025-06-09

**Authors:** Matthew Andrew, Sam Barraclough, James H. Dugdale, Matthew J. Reeves, Andrew O. Triggs, Adam L. Kelly

**Affiliations:** 1 Department of Sport and Exercise Sciences, Manchester Metropolitan University Institute of Sport, Manchester, United Kingdom; 2 School of Applied Sciences, Edinburgh Napier University, Edinburgh, United Kingdom; 3 Centre for Applied Sport, Physical Activity, and Performance, University of Central Lancashire, Preston, United Kingdom; 4 College of Sports Sciences & Physical Activity, Princess Nourah University, Riyadh, Saudi Arabia; 5 UCFB Manchester Campus, Manchester, United Kingdom; 6 Research Institute for Sport & Exercise Sciences, Liverpool John Moores University, Liverpool, United Kingdom; 7 Research for Athlete and Youth Sport Development (RAYSD) Lab, Faculty of Health, Education and Life Sciences, Birmingham City University, Birmingham, United Kingdom; Medical University of Warsaw: Warszawski Uniwersytet Medyczny, POLAND

## Abstract

Relative age effects in soccer are typified by an overrepresentation of players born earlier in the selection year. Examinations of relative age effects remain limited in female players and developing soccer nations. The aim of the present study was to examine the influence of sex, age, nation, playing position, and playing status in international level soccer players under Confederation of North, Central American and Caribbean Association Football (Concacaf). The sample consisted of a total of 1,959 active soccer players from 24 soccer nations that competed in recent Concacaf Championships. Results indicated an evident relative age effect in male [*p* < 0.05] but not female [*p* = 0.81] players. Male players were over‐represented by players born in the first quartile for the U17 [*p* < 0.01] level, however, this over‐representation did not transfer to the U20 or senior levels. No relative age effects were observed at any level for female players. A large proportion of nations demonstrated relative age effects in male, but not female samples. Relative age effects were shown for players participating at age group level, but not those ‘playing-up’. Results from this study highlight the continued disparity in relative age effects prevalence between male and female players raises further questions regarding the value of selecting relatively older players to metrics of success, transition, and selection for senior international soccer. This information can be used to advance talent identification and development in Concacaf nations.

## Introduction

Soccer continues to be one of the most popular female sports worldwide, with a significant increase in professionalisation and significant growth in both interest and participation over the last decade. For example, there has been a ~ 25% increase in the number of registered players between 2019–2023, rising to 16.6 million women and girls playing organised forms of the sport [1]. Equally, between 2016–2022 the Fédération Internationale de Football Association (FIFA) invested $2.8 billion across member associations and confederations, supporting initiatives to improve infrastructure, competitions, and stakeholder capacity for female soccer development [2]. This increased investment and popularity has provided nations with resources to aid talent identification (TID) and development (TDE) processes of youth players to ensure they receive appropriate support, coaching, and training across the pathway. As a result, nations attempt to identify (TID) female players with the potential to enter high-performance development programmes, with the aim of achieving future international success [3]. These processes are imperative at international level, as nations can typically only identify, develop, and (de)select from a pool of players that are eligible to represent the nation (i.e., they or their biological parent/grandparent were born in the nation). Indeed, female-specific TID studies remain limited, thus multiple calls have been made to further explore this area [4–7].

One of the most studied aspects surrounding TID is associated with the chronological age grouping of players for training and competition, which has been consistently highlighted in the literature for the past forty years, yet little has been done to attenuate these issues [8]. The use of birthdates as criterion for grouping players can lead to significant inter-individual differences in physical, psychological, and social characteristics within a single cohort, providing potential competitive advantages for chronologically older players. These (dis)advantages, known as ‘relative age effects’ (RAEs; [9,10]) can be characterised by an overrepresentation of players born closer to a ‘cut-off’ date compared to players born later in that same year. From a TID perspective, due to a combination of developmental advantages (advanced growth, older training age, impact of social agents), coaches/scouts may (sub)consciously judge relatively older players as more ‘talented’ [11,12]. Consequently, relatively younger players have demonstrated higher drop-out rates related with limited selection opportunities [20]. However, these advantages often dissipate or reverse at senior level [13–17] suggesting that it disrupts and potentially hinders long-term development and success.

Previous studies examining RAEs in male soccer have consistently illustrated an asymmetrical birth-date distribution in favour of relatively older players (e.g., [18,19]). In contrast, meta-analytical findings suggest an absence of RAEs, or smaller effects, in female populations compared to males [11,20]. For example, no RAEs were observed in 2,387 female players at U17, U20, and senior ages across the 55 European soccer nations [14]. Similarly, no RAEs were observed in senior female players from the top domestic league in Israel [21], or in youth female national team players from Switzerland and United States [22,23]. Contrastingly, other studies report small to medium effects for RAE prevalence in U17 and U19 international female players in Italy [24], RAEs at all domestic competition levels in Spanish female soccer [25], and all professional and international female players in Germany [26]. Recently, for example, Ribeiro et al. [27] investigated the prevalence of RAEs in a sample of 1,224 female soccer players who had participated in the U17, U20, and Senior FIFA Women’s Football World Cup. Results indicated no RAEs in the senior age category but identified significant RAEs for players in the U17 and U20 ages, with players born in Q1 being overrepresented.

There are a multitude of confounding factors that are known to influence TID and TDE processes between nations (e.g., talent pool population size, financial/logistical resources, strength of domestic competition, participation rates, number of coaches; [28,29]). Many studies examining the prevalence of RAEs in soccer contexts have predominantly been conducted within more established nations (e.g., [14,27], with only a limited number of studies have explored the prevalence of RAEs in lesser established nations, demonstrating contrasting findings in both youth female and male players (e.g., [30,31–33]). Recognising the potential disparity in characteristics between established and emerging soccer nations [28], further research is required to explore the recent prevalence of RAEs within different continental soccer confederations and the nations within them.

It must also be acknowledged that the RAEs can be moderated by certain factors such as playing position. For example, Ribeiro et al. [27] showed no RAEs across any playing position at the senior level, but significant RAEs for U17 and U20 midfielders. In contrast, Romann and Fuchslocher [22] demonstrated overall significant RAEs for defenders and goalkeepers in female Swiss soccer players aged between 10–20 years. Furthermore, another moderating factor that has recently received attention are from players deemed to possess higher levels of soccer-specific skills (e.g., technical, physical), and thus are invited to practice/compete at an age group above their own chronological age, commonly referred to as “playing-up” [34]. Analysis of RAEs in all 55 nations in the Union of European Football Nations (UEFA) from the most recent European championships revealed significant deviations in birth quartiles for males, but no evidence of RAEs in females who were playing-up [14]. It is suggested that allowing players to play up may account for some of the biases brought on by the prevalence of the RAE, providing appropriate developmental challenges for both earlier and later born players [17]. Whilst theoretically plausible, research merging these topics is absent in comprehensive samples of male and female soccer players from emerging football nations.

Accordingly, the aims of the present study were to examine the prevalence of RAEs in international female soccer players within the Confederation of North, Central American and Caribbean Association Football (Concacaf), which has the highest percentage (42.6%) of women and girls playing organised soccer compared to other federations [1]. Furthermore, the study aims to explore the influence of nation, age group, playing position, and playing status. To provide direct comparison, we will also examine RAEs in equivalent male players. Given the documented inconsistency of RAE prevalence in female soccer, it was hypothesised that a significant RAE bias would be observed in Concacaf male, but not female, players. Additionally, it was hypothesised that there would be further RAE biases across playing positions, in keeping with previous research (e.g., [35,36]). Given the limited research examining the influence of playing-up on RAE in soccer, we forgo making a priori hypotheses.

## Method

### Participants

Birthdates of 1,959 active Concacaf soccer players were obtained in November 2024 from the official data centres of Concacaf and individual nations. Birthdates were collected from 24 associations (out of 42) under the Concacaf governing body and from the rosters for the most recent Concacaf U17, U20, and senior Championships respectively (e.g., 2023 Men’s U17 Championships, Guatemala). All players were categorised by Sex: Male (929); Female (1030), Position: Goalkeeper (Male = 112; Female = 118); Defender (Male = 291; Female = 301); Midfielder (Male = 313; Female = 327); Forward (Male = 213; Female = 284), Age Group (i.e., age group): U17 (Male = 401; Female = 379;); U20 (Male = 252; Female = 398); Senior (Male = 276; Female = 253), and Playing Status (i.e., playing inside, or above their chronological age group): Age Group (male = 445; Female = 456); Playing-Up (Male = 208; Female = 321) (Table 1). Any players that were listed twice (e.g., making appearances at U17 and U20) were categorised based on the most appearances. Because data were freely available via the internet (www.concacaf.com), no approval by an ethical committee was required. The study was conducted in accordance with the declaration of Helsinki.

### Data analysis

The birth month for each player was used to define the birth quarter and semester distribution [18]. We adopted cut-off dates in line with Concacaf regulations, defined as: Q1 = January-March; Q2 = April-June; Q3 = July-September; Q4 = October-December, and semesters: S1 = January-June; S2 = July-December. The Chi-squared (χ^2^) test was used to assess differences between observed and expected birthdate distributions across quartiles and semesters for: (1) sex, irrespective of nation, playing position, age group, or playing status; (2) sex, age group, and position; (3) sex and playing status; and (4) sex and nation. Expected birthdates were obtained from an international database [37] and reflected the average population birthdate distributions for all available nations under Concacaf body competing in the respective championships (birthdates were not available for Grenada, Dominican Republic, Haiti, and Honduras) from 1984–2008, capturing the population records for birth years of the oldest and youngest players within the sample. Population birthdate distributions were identified as: Q1 = 24.1%; Q2 = 24.6%; Q3 = 26.3%; Q4 = 24.0%. Odds ratios (ORs) and 95% confidence intervals (95% CI) were calculated to compare the odds of the frequency of a quartile or semester to another with a reference group consisting of the relatively youngest players (Q4 or S2, respectively). An OR of 1.0 indicated that the frequency is equal in both quarters/semesters whilst an OR of 2.0 indicated that the frequency of one quarter/semester is twice as high as the other [20,26]. ORs were considered significant if the 95% CI range did not include a value <1.00. Moreover, effect sizes (Cohen’s W) were calculated to determine the magnitude of chi‐squared tests [38], proposed that where *w* = 0.10, *w* = 0.30, and *w* = 0.50, they specified small, medium, and large effect sizes, respectively. Where appropriate, alpha was set at *p* < 0.05. Data were analysed via SPSS Statistics Version 26.0 for Windows (IBM, Chicago USA).

## Results

**Fig 1:**
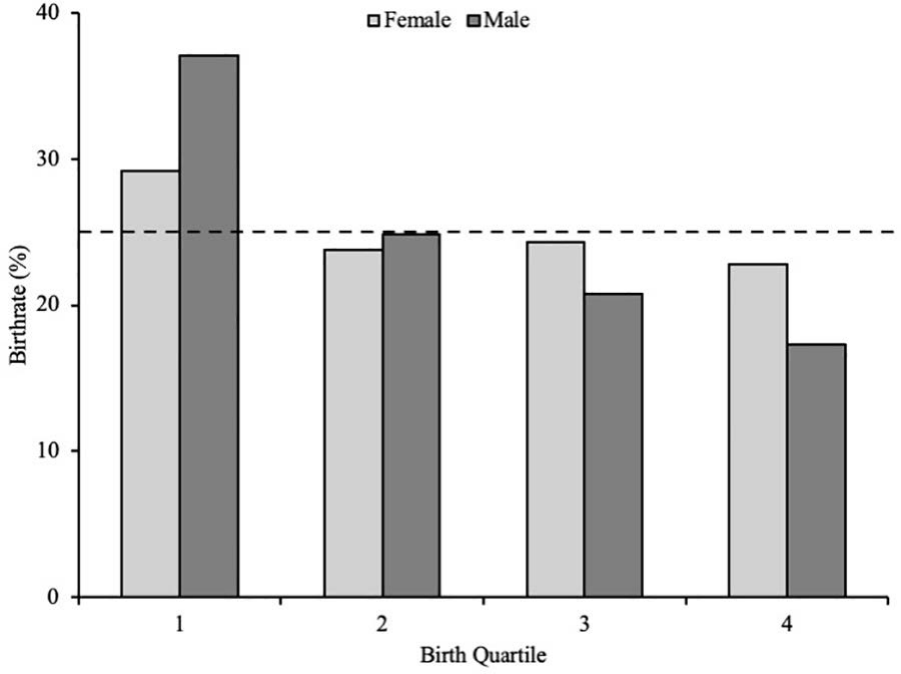
Birth quartile distribution for male (light grey) and female (dark grey) soccer players.

There were statistically significant RAEs for male players [χ2 = (*n* = 929) = 10.65, *p* < 0.05, *w* = 0.11], but not female players [χ2 = (*n* = 1030) = 1.44, *p* = 0.70, *w* = 0.04]. Male players born in the first quartile were over‐represented (Q1 vs. Q4, OR = 2.1, CI = 0.9–4.6, Fig 1), and the ORs declined marginally for comparisons later in the year, with Q4 being inferior for each.

### Age group and playing position

The frequency and percentage distributions of players’ birth quartiles for age group and playing position status are presented in Table 2. For age group, irrespective of playing position, there were statistically significant RAEs at U17 level for males [χ2 = (*n* = 401) = 23.54, *p* < 0.001, *w* = 0.24] but not female players [χ2 = (*n* = 379) = 2.54, *p* = 0.48, *w* = 0.08]. In female U17 and senior players there were significant deviations in birth quartiles for goalkeepers [U17: χ2 = (*n* = 43) = 8.35, *p* < 0.05, *w* = 0.44; senior: χ2 = (*n* = 31) = 13.63, *p* < 0.05, *w* = 0.66], defenders [U17: χ2 = (*n* = 102) = 9.77, *p* < 0.05, *w* = 0.31], and midfielders [U17: χ2 = (*n* = 126) = 10.38, *p* < 0.05, *w* = 0.29]. For senior goalkeepers, players born in the first quartile (32.3%) being over‐represented (Q1 vs. Q4: OR = 3.2, CI = 1.3–8.0) with Q4 (9.7%) being inferior. For U17 defender, players born in the third quartile (33.3%) being over‐represented (Q3 vs. Q4: OR = 2.1, CI = 0.9–4.7), with Q4 (16.7%) being inferior. Whereas for U17 midfielders, the largest distribution was Q4 (34.1%), with Q3 being inferior (14.3%). In male players, U17 and U20 players indicated significant deviations in birth quartiles for goalkeepers [U17: χ2 = (*n* = 40) = 30.53, *p* < 0.001, *w* = 0.87; U20: χ2 = (*n* = 36) = 9.70, *p* < 0.05, *w* = 0.52] defenders [U17: χ2 = (*n* = 128) = 20.45, *p* < 0.001, *w* = 0.40; U20: χ2 = (*n* = 71) = 13.34, *p* < 0.05, *w* = 0.43], midfielders [U17: χ2 = (*n* = 138) = 19.82, *p* < 0.001, *w* = 0.38], and forwards [U17: χ2 = (*n* = 95) = 32.74, *p* < 0.001, *w* = 0.59; U20: χ2 = (*n* = 64) = 12.49, *p* < 0.01, *w* = 0.44], with players born in the first quartile (goalkeepers: U17 = 45.0%; defenders: U17 = 42.2%; U20 = 32.4%; midfielders: U17 = 42.0%; forwards: U17 = 46.3%; U20 = 39.1%) being over‐represented (Q1 vs Q4, goalkeepers; U17: OR = 2.9, CI = 1.3–6.5; defenders; U17: OR = 3.1, CI = 1.3–7.0; U20: OR = 3.2, CI = 1.3–7.8; midfielders; U17: OR = 2.8, CI = 1.2–6.3; forwards; U17: OR = 4.7, CI = 1.9–11.5; U20: OR = 2.0, CI = 0.9–4.4), with Q4 (goalkeepers: U17 = 15.0%; defenders: U17 = 13.3%; U20 = 9.9%; midfielders: U17 = 14.5%; forwards: U17 = 9.5%; U20 = 18.8%) being lesser for each case. For U20 goalkeepers, players born in the second quartile (36.1%) were over-represented compared to quartile three (16.7%).

**Table 1:** Categories, subcategories, and definitions of independent variables.

Category	Subcategory	Definition
Sex	Female	Represented their respective nations female soccer team.
	Male	Represented their respective nations male soccer team.
Age Group	U17	Represented their nation during the U17 Concacaf Championships.
	U20	Represented their nation during the U20 Concacaf Championships.
	Senior	Represented their nation during the senior Concacaf Championships.
Playing Status	Age Group	Playing inside their age group (e.g., 16/17 years playing at U17 level).
	Playing-Up	Playing above their age group (e.g., 17/18 years playing at U20 level)

**Table 2:** Birth quartile distribution by sex, age group, and playing position (* Significant at an alpha level of *p* < 0.05).

				Birthdate Distribution (%)				Odds Ratio (95% CI)						
	Age	Position	*n*	Q1	Q2	Q3	Q4	Q1 vs. Q4	Q2 vs. Q4	Q3 vs. Q4	S1 vs. S2	*X* ^*2*^	*p*	*W*
Male	U17	Goalkeeper	40	18 (45)	11 (28)	5 (13)	6 (15)	2.9 (1.3-6.5)	1.8 (0.8-4.2)	0.9 (0.3-2.2)	2.6 (1.5-4.7)	30.53	<0.001	0.87
		Defender	128	54 (42)	30 (23)	27 (21)	17 (13)	3.1 (1.3-7.0)	1.7 (0.7-4.2)	1.7 (0.7-4.0)	1.9 (1.1-3.4)	20.45	<0.001	0.40
		Midfielder	138	58 (42)	33 (24)	27 (20)	20 (15)	2.8 (1.2-6.3)	1.6 (0.7-3.8)	1.4 (0.6-3.4)	1.9 (1.1-3.4)	19.82	<0.001	0.38
		Forward	95	44 (46)	24 (25)	18 (19)	9 (10)	4.7 (1.9-11.5)	2.6 (1.6-7.0)	2.1 (0.8-5.5)	2.5 (1.4-4.5)	32.74	<0.001	0.59
		TOTAL	401	174 (43)	98 (24)	77 (19)	52 (13)	3.2 (1.4-7.4)	1.9 (0.8-4.4)	1.6 (0.6-3.8)	2.1 (1.2-3.7)	23.54	<0.001	0.24
	U20	Goalkeeper	36	9 (25)	13 (36)	6 (17)	8 (22)	1.1 (0.5-2.4)	1.6 (0.7-3.4)	0.8 (0.3-1.8)	1.6 (0.9-2.8)	9.70	<0.050	0.52
		Defender	71	23 (32)	20 (28)	21 (30)	7 (10)	3.2 (1.3-7.8)	2.8 (1.1-7.0)	3.2 (1.3-7.8)	1.5 (0.9-2.7)	13.34	<0.005	0.43
		Midfielder	81	28 (35)	19 (24)	18 (22)	16 (20)	1.7 (0.8-3.7)	1.2 (0.5-2.6)	1.2 (0.5-2.7)	1.4 (0.8-2.4)	6.43	0.1000	0.28
		Forward	64	25 (39)	13 (20)	14 (22)	12 (19)	2.0 (0.9-4.4)	1.1 (0.5-2.5)	1.2 (0.5-2.8)	1.5 (0.8-2.6)	12.49	<0.010	0.44
		TOTAL	252	85 (34)	65 (26)	59 (23)	43 (17)	1.9 (0.8-4.3)	1.5 (0.7-3.4)	1.4 (0.6-3.3)	1.5 (0.8-2.6)	6.87	0.080	0.17
	Senior	Goalkeeper	36	12 (33)	9 (25)	6 (17)	9 (25)	1.3 (0.6-2.8)	1.0 (0.4-2.2)	0.7 (0.3-1.6)	1.4 (0.8-2.4)	7.39	0.070	0.45
		Defender	92	32 (35)	23 (25)	20 (22)	17 (19)	1.8 (0.8-4.0)	1.3 (0.6-3.0)	1.2 (0.5-2.8)	1.5 (0.8-2.6)	7.37	0.060	0.28
		Midfielder	94	27 (29)	22 (23)	20 (21)	25 (27)	1.0 (0.5-2.2)	0.9 (0.4-1.9)	0.8 (0.4-1.9)	1.1 (0.6-1.9)	2.10	0.570	0.15
		Forward	54	14 (26)	14 (26)	11 (20)	15 (28)	0.9 (0.4-2.0)	0.9 (0.4-2.0)	0.8 (0.3-1.7)	1.1 (0.6-1.9)	1.99	0.600	0.19
		TOTAL	276	85 (31)	68 (25)	57 (21)	66 (24)	1.2 (0.6-2.7)	1.0 (0.5-2.2)	0.9 (0.4-2.0)	1.2 (0.7-2.2)	3.23	0.370	0.11
Female	U17	Goalkeeper	43	9 (21)	14 (33)	7 (16)	13 (30)	0.7 (0.3-1.5)	1.1 (0.5-2.2)	0.6 (0.3-1.3)	1.2 (0.7-2.0)	8.35	<0.050	0.44
		Defender	102	33 (32)	18 (18)	34 (33)	17 (17)	1.9 (0.8-4.2)	1.0 (0.4-2.5)	2.1 (0.9-4.7)	1.0 (0.6-1.7)	9.77	<0.050	0.31
		Midfielder	126	36 (29)	29 (23)	18 (14)	43 (34)	0.8 (0.4-1.7)	0.7 (0.3-1.4)	0.4 (0.2-1.0)	1.1 (0.6-1.9)	10.38	<0.050	0.29
		Forward	108	35 (32)	22 (20)	24 (22)	27 (25)	1.2 (0.6-2.7)	0.8 (0.4-1.8)	0.9 (0.4-2.1)	1.1 (0.6-1.9)	4.30	0.240	0.20
		TOTAL	379	113 (30)	83 (22)	83 (22)	100 (26)	1.1 (0.5-2.3)	0.8 (0.4-1.8)	0.9 (0.4-1.9)	1.1 (0.6-1.9)	2.54	0.480	0.08
	U20	Goalkeeper	44	14 (32)	10 (23)	13 (30)	7 (16)	1.9 (0.8-4.4)	1.4 (0.6-3.3)	2.0 (0.9-4.4)	1.2 (0.7-2.1)	6.48	0.100	0.38
		Defender	113	35 (31)	25 (22)	29 (26)	24 (21)	1.4 (0.6-3.1)	1.0 (0.5-2.3)	1.3 (0.6-2.8)	1.1 (0.6-2.0)	2.82	0.430	0.16
		Midfielder	122	31 (25)	36 (30)	27 (22)	28 (23)	1.1 (0.5-2.4)	1.3 (0.6-2.8)	1.0 (0.5-2.3)	1.2 (0.7-2.1)	1.97	0.600	0.13
		Forward	119	31 (26)	25 (21)	35 (29)	28 (24)	1.1 (0.5-2.4)	0.9 (0.4-2.0)	1.3 (0.6-2.8)	0.9 (0.5-1.5)	1.18	0.770	0.10
		TOTAL	398	111 (28)	96 (24)	104 (26)	87 (22)	1.2 (0.6-2.7)	1.1 (0.5-2.4)	1.3 (0.6-2.8)	1.1 (0.6-1.9)	1.02	0.800	0.05
	Senior	Goalkeeper	31	10 (32)	9 (29)	9 (29)	3 (10)	3.2 (1.3-8.0)	3.0 (1.2-7.4)	3.2 (1.3-7.8)	1.6 (0.9-2.8)	13.63	<0.005	0.66
		Defender	86	26 (30)	20 (23)	17 (20)	23 (27)	1.1 (0.5-2.3)	0.9 (0.4-1.9)	0.8 (0.3-1.7)	1.2 (0.7-2.0)	3.53	0.340	0.20
		Midfielder	79	22 (28)	20 (25)	25 (32)	12 (15)	1.8 (0.8-4.1)	1.6 (0.7-3.8)	2.2 (1.0-5.0)	1.1 (0.7-2.0)	5.78	0.140	0.27
		Forward	57	18 (32)	17 (30)	12 (21)	10 (18)	1.7 (0.8-3.9)	1.7 (0.7-3.8)	1.3 (0.5-2.9)	1.6 (0.9-2.8)	6.90	0.080	0.35
		TOTAL	253	76 (30)	66 (26)	63 (25)	48 (19)	1.5 (0.7-3.4)	1.4 (0.6-3.0)	1.4 (0.6-3.1)	1.3 (0.7-2.2)	3.14	0.380	0.11

### Soccer nation

The frequency and percentage distributions of players’ birth quartiles for sex and nation are presented in Table 3. In female players, there were statistically significant RAEs for Bermuda, Costa Rica, Dominican Republic, El Salvador, Grenada, Guatemala, Haiti, Mexico, Panama, and Suriname. For these nations, OR analysis indicated that players born in Q1 were overrepresented with Q4 being inferior in each case. However, for Grenada and Suriname, the largest distribution was Q2, with Q3 being inferior for Grenada, and Q1 and Q4 for Suriname. For Dominican Republic and Panama, the largest distribution was Q3, with Q2 being inferior for the Dominican Republic and Q4 for Panama. For Costa Rica, the largest distribution was Q4, with Q2 being inferior. For male players, there were statistically significant RAEs for Canada, Costa Rica, Cuba, Curaçao, Dominican Republic, El Salvador, Guadeloupe, Jamaica, Nicaragua, Panama, Puerto Rico, Suriname, and Trinidad and Tobago.

**Table 3:** Birth quartile distribution by nation and sex (* Significant at an alpha level of *p* < 0.05).

			Birthdate Distribution (%)				Odds Ratio (95% CI)						
Nation	Sex	*n*	Q1	Q2	Q3	Q4	Q1 vs. Q4	Q2 vs. Q4	Q3 vs. Q4	S1 vs. S2	*X* ^*2*^	*p*	*W*
Barbados	Male	13	3 (23)	4 (31)	3 (23.1)	3 (23)	1.0 (0.4-2.2)	1.3 (0.6-2.8)	1.1 (0.5-2.3)	1.2 (0.7-2.0)	2.21	0.550	0.41
	Female	–	–	–	–	–	–	–	–	–	–	–	–
Bermuda	Male	13	4 (31)	4 (31)	3 (23)	2 (15)	1.9 (0.8-4.4)	2.0 (0.9-4.5)	1.6 (0.7-3.7)	1.6 (0.9-2.8)	7.69	0.060	0.77
	Female	40	15 (38)	7 (18)	13 (33)	5 (13)	2.9 (1.2-6.8)	1.4 (0.6-3.4)	2.7 (1.2-6.4)	1.2 (0.7-2.1)	17.62	<0.001	0.66
Canada	Male	67	24 (36)	18 (27)	13 (19)	12 (18)	1.9 (0.9-4.3)	1.5 (0.7-3.3)	1.1 (0.5-2.7)	1.7 (1.0-3.0)	9.97	<0.05	0.39
	Female	63	20 (32)	16 (25)	14 (22)	13 (21)	1.5 (0.7-3.3)	1.2 (0.5-2.7)	1.1 (0.5-2.6)	1.3 (0.8-2.3)	3.93	0.280	0.25
Cayman Islands	Male	–	–	–	–	–	–	–	–	–	–	–	–
	Female	20	4 (20)	5 (25)	6 (30)	5 (25)	0.8 (0.3-1.7)	1.0 (0.4-2.2)	1.3 (0.6-2.7)	0.8 (0.5-1.4)	1.27	0.750	0.25
Costa Rica	Male	66	27 (41)	19 (29)	15 (23)	5 (8)	5.2 (2.0-13.5)	3.7 (1.4-9.9)	3.2 (1.2-8.5)	2.3 (1.3-4.1)	25.58	<0.001	0.62
	Female	43	10 (23)	4 (9)	10 (23)	19 (44)	0.5 (0.2-1.1)	0.2 (0.1-0.5)	0.6 (0.3-1.2)	0.5 (0.3-0.9)	25.4	<0.001	0.77
Cuba	Male	54	25 (46)	10 (19)	12 (22)	7 (13)	3.4 (1.5-7.9)	1.4 (0.6-3.5)	1.8 (0.8-4.3)	1.8 (1.0-3.2)	28.68	<0.001	0.73
	Female	40	8 (20)	10 (25)	10 (25)	12 (30)	0.6 (0.3-1.4)	0.8 (0.4-1.8)	0.9 (0.4-1.9)	0.8 (0.5-1.4)	1.81	0.620	0.21
Curaçao	Male	25	11 (44)	7 (28)	3 (12)	4 (16)	2.7 (1.2-5.9)	1.7 (0.8-3.9)	0.8 (0.3-2.0)	2.6 (1.4-4.6)	28.76	<0.001	1.07
	Female	40	11 (28)	9 (23)	10 (25)	10 (25)	1.1 (0.5-2.3)	0.9 (0.4-2.0)	1.1 (0.5-2.3)	1.0 (0.6-1.7)	0.73	0.870	0.14
Dominican Rep	Male	39	20 (51)	5 (13)	8 (21)	6 (15)	3.2 (1.4-7.1)	0.8 (0.3-2.1)	1.4 (0.6-3.3)	1.8 (1.0-3.1)	41.64	<0.001	1.03
	Female	63	17 (27)	10 (16)	24 (38)	12 (19)	1.4 (0.6-3.1)	0.8 (0.3-2.0)	2.1 (1.0-4.6)	0.8 (0.4-1.3)	10.75	<0.05	0.41
El Salvador	Male	66	24 (36)	19 (29)	14 (21)	9 (14)	2.6 (1.1-5.9)	2.1 (0.9-4.9)	1.6 (0.7-3.9)	1.9 (1.1-3.3)	13.4	<0.005	0.45
	Female	43	16 (37)	11 (26)	11 (26)	5 (12)	3.1 (1.3-7.3)	2.2 (0.9-5.3)	2.3 (1.0-5.6)	1.7 (1.0-3.0)	14.61	<0.005	0.58
Grenada	Male	–	–	–	–	–	–	–	–	–	–	–	–
	Female	20	5 (25)	7 (35)	3 (15)	5 (25)	1.0 (0.4-2.1)	1.4 (0.6-2.9)	0.6 (0.3-1.5)	1.5 (0.9-2.6)	9.82	<0.05	0.70
Guadeloupe	Male	31	12 (39)	8 (26)	6 (19)	5 (16)	2.3 (1-5.2.0)	1.6 (0.7-3.6)	1.3 (0.5-3.0)	1.8 (1.0-3.2)	14.18	<0.005	0.68
	Female	–	–	–	–	–	–	–	–	–	–	–	–
Guatemala	Male	53	15 (28)	13 (25)	13 (25)	12 (23)	1.2 (0.5-2.6)	1.1 (0.5-2.4)	1.1 (0.5-2.5)	1.1 (0.6-2.0)	1.09	0.780	0.14
	Female	63	23 (37)	20 (32)	9 (14)	11 (18)	2.0 (0.9-4.5)	1.8 (0.8-4.0)	0.9 (0.4-2.1)	2.2 (1.2-3.8)	16.86	<0.005	0.52
Guyana	Male	–	–	–	–	–	–	–	–	–	–	–	–
	Female	63	22 (35)	15 (24)	12 (19)	14 (22)	1.5 (0.7-3.3)	1.1 (0.5-2.4)	0.9 (0.4-2.0)	1.4 (0.8-2.5)	7.39	0.070	0.34
Haiti	Male	72	25 (35)	18 (25)	13 (18)	16 (22)	1.5 (0.7-3.3)	1.1 (0.5-2.5)	0.9 (0.4-2.0)	1.5 (0.8-2.6)	7.83	0.060	0.33
	Female	63	24 (38)	8 (13)	13 (21)	18 (29)	1.3 (0.6-2.7)	0.4 (0.2-1.0)	0.8 (0.3-1.7)	1.0 (0.6-1.8)	15.87	<0.005	0.50
Honduras	Male	70	18 (26)	18 (26)	21 (30)	13 (19)	1.3 (0.6-3.0)	1.4 (0.6-3.1)	1.7 (0.8-3.8)	1.1 (0.6-1.8)	2.44	0.510	0.19
	Female	40	10 (25)	10 (25)	7 (18)	13 (33)	0.7 (0.3-1.6)	0.8 (0.4-1.6)	0.6 (0.3-1.3)	1.0 (0.6-1.7)	5.59	0.160	0.37
Jamaica	Male	64	19 (30)	15 (23)	8 (13)	22 (34)	0.8 (0.4-1.8)	0.7 (0.3-1.4)	0.4 (0.2-0.9)	1.1 (0.7-2.0)	12.9	<0.01	0.45
	Female	40	13 (33)	9 (23)	8 (20)	10 (25)	1.3 (0.6-2.7)	0.9 (0.4-2.0)	0.8 (0.4-1.9)	1.2 (0.7-2.1)	4.76	0.200	0.34
Mexico	Male	67	23 (34)	18 (27)	15 (22)	11 (16)	2.0 (0.9-4.5)	1.6 (0.7-3.7)	1.4 (0.6-3.3)	1.6 (0.9-2.8)	8.25	<0.05	0.35
	Female	63	22 (35)	17 (27)	16 (25)	8 (13)	2.7 (1.1-6.2)	2.1 (0.9-5.0)	2.1 (0.9-5.0)	1.6 (0.9-2.9)	11.41	<0.05	0.43
Nicaragua	Male	24	13 (54)	4 (17)	4 (17)	3 (13)	4.2 (1.8-9.6)	1.3 (0.5-3.3)	1.4 (0.6-3.5)	2.4 (1.4-4.3)	50.46	<0.001	1.45
	Female	40	11 (28)	12 (30)	6 (15)	11 (28)	1.0 (0.4-2.1)	1.1 (0.5-2.3)	0.6 (0.2-1.3)	1.4 (0.8-2.4)	7.25	0.080	0.43
Panama	Male	67	28 (42)	18 (27)	11 (16)	10 (15)	2.7 (1.2-6.1)	1.8 (0.8-4.1)	1.2 (0.5-2.8)	2.2 (1.2-3.9)	21.46	<0.001	0.57
	Female	63	17 (27)	12 (19)	26 (41)	8 (13)	2.0 (0.9-4.9)	1.5 (0.6-3.6)	3.4 (1.5-7.9)	0.9 (0.5-1.5)	17.2	<0.005	0.52
Puerto Rico	Male	18	11 (61)	4 (22)	1 (6)	2 (11)	5.3 (2.3-12.4)	2.0 (0.8-4.9)	0.5 (0.2-1.7)	5.0 (2.6-9.6)	82.93	<0.001	2.15
	Female	63	21 (33)	15 (24)	15 (24)	12 (19)	1.7 (0.8-3.7)	1.2 (0.5-2.8)	1.3 (0.6-3)	1.3 (0.8-2.3)	5.29	0.160	0.29
St. Kitts and Nevis	Male	–	–	–	–	–	–	–	–	–	–	–	–
	Female	37	6 (16)	9 (24)	9 (24)	13 (35)	0.4 (0.2-1)	0.7 (0.3-1.5)	0.7 (0.3-1.5)	0.7 (0.4-1.2)	7.01	0.080	0.44
Suriname	Male	12	3 (25)	1 (8)	6 (50)	2 (17)	1.4 (0.6-3.3)	0.5 (0.2-1.3)	3.2 (1.4-6.9)	0.5 (0.3-0.9)	37.2	<0.001	1.76
	Female	20	3 (15)	8 (40)	6 (30)	3 (15)	1.0 (0.4-2.4)	2.6 (1.2-5.9)	2.1 (0.9-4.8)	1.2 (0.7-2.1)	17.99	<0.001	0.95
Trinidad and Tobago	Male	48	20 (42)	12 (25)	8 (17)	8 (17)	2.4 (1.1-5.3)	1.5 (0.6-3.4)	1.1 (0.4-2.5)	2.0 (1.1-3.5)	19.54	<0.001	0.64
	Female	40	7 (18)	11 (28)	8 (20)	14 (35)	0.5 (0.2-1.1)	0.8 (0.4-1.6)	0.6 (0.3-1.3)	0.8 (0.5-1.4)	7.95	0.050	0.45
United States	Male	60	19 (32)	16 (27)	16 (27)	9 (15)	2.0 (0.9-4.7)	1.7 (0.8-4.1)	1.9 (0.8-4.3)	1.4 (0.8-2.4)	6.71	0.090	0.33
	Female	63	15 (24)	20 (32)	14 (22)	14 (22)	1.0 (0.5-2.3)	1.4 (0.6-3.1)	1.1 (0.5-2.3)	1.3 (0.7-2.2)	3.13	0.390	0.22

### Playing status

The frequency and percentage distributions of players’ birth quartiles for sex and playing status are presented in Table 4. There was a statistically significant RAE for male players at age group [χ2 = (*n* = 445) = 33.83, *p* < 0.001, *W* = 0.28], but not for playing-up [χ2 = (*n* = 208) = 3.87, *p* = 0.30, *w* = 0.14] or female players [Age Group: χ2 = (*n* = 456) = 5.28, *p* = 0.16, *w* = 0.11; Playing-Up: χ2 = (*n* = 321) = 0.56, *p* = 0.91, *w* = 0.04]. Male Age Group players born in the first quartile (46.0%) were over‐represented (Q1 vs Q4, OR = 4.0, CI = 1.7–9.6), with Q4 (11.0%) being inferior.

**Table 4:** Birth quartile distribution by sex and playing status (* Significant at an alpha level of *p* < 0.05).

				Birthdate Distribution (%)				Odds Ratio (95% CI)						
Sex	Playing Status		*n*	Q1	Q2	Q3	Q4	Q1 vs. Q4	Q2 vs. Q4	Q3 vs. Q4	S1 vs. S2	*X* ^*2*^	*p*	*W*
Male	Age Group		445	205 (46)	124 (28)	67 (15)	49 (11)	4.0 (1.7-9.6)	2.5 (1.0-6.1)	1.4 (0.6-3.7)	2.8 (1.6-5.1)	33.83	<0.001	0.28
	Playing-Up		208	54 (26)	39 (19)	69 (33)	46 (22)	1.1 (0.5-2.5)	0.8 (0.4-1.9)	1.6 (0.7-3.4)	0.8 (0.5-1.4)	3.87	0.300	0.14
Female	Age Group		456	154 (34)	92 (20)	106 (23)	104 (23)	1.4 (0.7-3.1)	0.9 (0.4-2.0)	1.1 (0.5-2.4)	1.2 (0.7-2.0)	5.28	0.160	0.11
	Playing-Up		321	70 (22)	87 (27)	81 (25)	83 (26)	0.8 (0.4-1.8)	1.0 (0.5-2.2)	1.0 (0.5-2.2)	1.0 (0.5-1.7)	0.56	0.910	0.04

## Discussion

The aim of the present study was to investigate the prevalence of RAEs in a comprehensive sample of multi-national female and male soccer players within Concacaf, considering sex, nationality, age group, playing position, and playing status. Our main findings were: (1) limited RAEs were observed in U17, U20, and Senior female Concacaf players; (2) medium to large RAE biases were found within almost all playing positions for male youth U17 and U20 players, which dissipated at the Senior level; (3) statistically significant RAEs were observed for male youth players playing at age group level, but not for those playing-up; and, (4) uneven birthdate distributions were observed in 11/20 nations for males compared to 10/22 nations for female players. Analysis of the total samples for both male and female players demonstrated a statistically significant deviation in birthdate distributions in male, but not female Concacaf soccer players who demonstrated relatively equal birthdate distributions (Fig 1). Equally, when considering age group, no RAEs were present for females in any age categories.

It has been suggested that an absence of RAEs in female soccer may be due to a reduced popularity and participation of the sport in comparison to males [26]. However, a recent FIFA report demonstrated women and girls playing organised soccer is the highest percentage (42.6%) under Concacaf compared to other confederations [1]. Increased participation has previously been reported as a possible causal condition for RAEs, whereby increased participation leads to greater competition for places [39]. However, despite this increased participation, the access to competitive opportunities within female soccer is still far less than for males. This depth of competition hypothesis [40,41] suggests that with more players competing for a finite number of places, the more likely the characteristics of relatively older youth will be valued, leading to potentially stronger RAEs. This may exacerbate the issue of stakeholders (i.e., scouts/coaches) equating ‘talent’ with current performance, with relatively older players more likely to benefit due to the perceived physical and psychosocial advantages associated with having an increased chronological and developmental training age [8]. However, our findings conflict with this, with limited evidence of RAEs in female soccer players within our sample. This may partly be due to a large majority of Concacaf nations having less established infrastructure and sport selection programmes despite high participation rates, leading to a reduced talent pool for selection [28,39]. Equally, when considering the social aspects of participation, historic sociocultural norms may impact rates of drop-out and retention. This may particularly be the case in early maturing female players, where the physical requirements of athletic success and the associated morphological adaptations, may conflict with gender-based stereotypes of ideal body images, leading to increased rates of drop out during periods of maturation [22,42], further reducing the talent pool.

From a male perspective, similar to other national youth team samples (e.g., [14,32,43,44]), significant RAEs were present within the U17 age group, but then dissipate at senior level (Table 2). These findings are consistent with contemporary results of longitudinal RAE research, which suggests earlier selection into soccer TD pathways exacerbates RAEs in younger age groups [45] and may cause a cascading effect whereby early identification and (de)selection processes continue to re-bias selections in favour of chronologically older players [13,14,44]. Equally, longitudinal analysis has revealed relatively younger players are more likely to be selected at senior levels [44,46] suggesting a possible ‘reversal’ of RAEs [15,24,47]. The reduced prevalence of RAEs in senior age groups may be related to the distinct concepts of RAEs and biopsychosocial maturation [48,49]. For example, in youth and adolescent cohorts biological maturation, psychological development and socio-emotional factors can vary dramatically between individuals leading to a range of associated (dis)advantages and challenges [50], with these (dis)advantages becoming attenuated during adulthood as a result of a reduction in experience and opportunities to practice between relatively older and younger players [44].

When exploring positional differences in RAEs (Table 2), it was observed that all positions for youth males (U17; U20) had asymmetrical birthdate distributions, except for midfield players at U20. Previous examinations of the role of playing position have reported that RAEs are most prevalent in goalkeepers and defenders [23,35,51–53]. It has been suggested that anthropometrics and physical skills that are required to meet the demands of the game may play a role in RAEs [32], which has been shown in other sports such as rugby [54], handball [55], and hockey [56]. Furthermore, the lack of RAE in U20 midfielders may be associated with attacking positions emphasising technical over physical skills [57]. However, it is important to acknowledge that we did not collect anthropometrics or physical data, and we only provided four positions. In contrast, the lack of observed RAEs across positions within the majority of the youth female samples in this study may suggest that within Concacaf nations, talented female soccer players are identified and selected for youth national teams based on skill-based (e.g., technical, perceptual-cognitive) or sociological (e.g., hours of practice) predictors of high performance [3], rather than competitive advantages associated with developmental differences due to increased age and experience [40]. In line with Finnegan et al. [23], a significant difference in birthdates was observed in senior female goalkeepers, which has been suggested to be attributed to a preference for ‘taller’ players [28]. As per our findings, these authors indicated this may be partially explained by the relatively small sample size for this position rather than the anthropometric and physical attributes typically expected for that position, which may have been more salient in earlier-born players at youth levels [14,22,25].

Our results also demonstrated no differences in birthdate distributions for females playing at age group ‘level’ or ‘playing-up’ (Table 4), in keeping with both youth and senior female national teams in Europe [14]. Kelly et al. [17] suggested that the idea of playing-up may moderate the RAE by introducing a new quota of relatively younger players. This may also create an ‘underdog’ effect providing a challenge for those who play up to overcome the associated disadvantages presented to them by the RAE [17,47,58]. For male players in our study, birthdate distributions differed at the age group level but not for those playing-up. This contrasts with Kelly et al. [17], who found that over 70% of U12-U16 players playing up were born in the first half of the year, in addition to having superior technical, tactical, psychological, and social traits. However, in our multinational sample, relatively older players were not favoured for playing up. Interestingly, although overall differences were not found, when examining individual age groups (i.e., U17; U20), differences in birthdate distributions were found for those playing-up at U17 but not U20 level, suggesting some relative age-related differences in physicality, experience, and development can be advantageous in being selected to participate in older age groups.

When observing RAEs by individual nation, a larger proportion of countries favoured earlier born players amongst male rather than female players (Table 3). Whilst small sample sizes may have impacted results for some nations in male samples (e.g., Bermuda; Puerto Rico; Suriname), the presence of RAEs in some nations amongst the female samples contrast with the wider results we have previously reported. Many nations within Concacaf may be characterised as emerging football nations who are purported to have lower participation rates, and less financial and logistical resources [28]. Thus, the prevalence of RAEs (or lack thereof), may be partly due to the relative size of the talent pool from which nations can select. However, this is difficult to interpret without in-depth knowledge of the cultural, structural, and societal factors that affect TID and TDE philosophies and processes within individual nations. Further information and research are required to understand the variability of these factors between different nations and the subsequent impact on TID processes. From a societal viewpoint, socio-economic status can lead to differences in RAE. Players in nations with higher socio-economic status are likely to have greater access to facilities, qualified coaches, and talent development pathways which may reduce the RAE bias [59], with nations of lower socio-economic status having restricted resources and opportunities having more pronounced RAEs in youth soccer [60]. Families of greater means can provide greater levels of financial and/or emotional support to players and thus mitigate the disadvantages of RAE. For example, female soccer in the United States is characterised by a “pay-to-play” model, which results in a player’s access to learning environment is underpinned by their parental financial support and sociocultural and economic status [46]

Given the continued existence of RAEs in youth soccer, researchers have attempted to provide potential practical strategies that could be implemented in sport selection systems to reduce these biases [61]. For example, it has previously been shown that placing numbers on players shirts that correspond to their relative age or biological maturation status results in scouts being more likely to provide higher ratings to younger and later maturing players [62,63]. Moreover, it has been suggested that players are grouped by maturity status rather than chronological age, also referred to as “bio-banding” [64]. This method is suggested to be more sensitive to individual differences in size and athleticism associated with biological maturation, thus creating more suitable settings for practice and competition where less mature players can be evaluated based on their technical, tactical, and psychological skills without more mature players relying on their temporary physical advantages [65–69]. Further, there may be a need to introduce contemporary methodological approaches (e.g., [70]) to standardise RAE measures and reduce methodological biases across different sports, and within analyses adopting different age category boundaries and cut-off dates (e.g., age groups spanning two-, three-, or four-years). Introducing such methods may reduce some of the deficiencies demonstrated in research using samples with different grouping methods [11] and permit cross-cohort and sport comparisons of RAEs and constituent year effects [70–72]. Whilst providing promising empirical evidence to reduce RAEs, studies using some of these methodologies highlighted above are still in their infancy and the long-term effects of these practices are currently unclear. However, researchers should continue in their efforts to mitigate the effects of RAEs in youth sports.

## Limitations

It must be acknowledged that this study is not without limitations. As only birthdate, sex, and nationality data were extracted for participants from external sources, we did not obtain any data relating to players technical, psychological, or physical skills [73]. Therefore, any suggestions of selected players having superior sport-specific skills are based upon theoretical reasoning rather than empirical proof. For example, research has demonstrated that biological maturation, rather than relative age, may be the primary factor in physical and physiological advantages for youth athletes [12,74] and could also heavily influence selection processes (e.g., [49,75,76]). Considering this, research has suggested it may be unwise to continue to examine RAEs in cross-sectional, exploratory contexts [8]. Moreover, we classified four playing positions. A more nuanced classification by differentiating between wide/central positions [23] could potentially provide further insights related to the influence of the anthropometrical and physical demands of different playing positions on RAEs. However, the ﬁndings from this study provide a comprehensive and novel exploration of RAEs in male and female players, providing insight into TID processes across multiple emerging soccer nations. Future research should consider employing longitudinal research designs and explore youth to senior transitions, whilst accounting for individual differences to identify potential variances of RAEs within selected Concacaf players and propose potential solutions to mitigate such effects.

## Conclusion

Our findings demonstrate that RAEs exists in male but not female soccer players participating in Concacaf tournaments between 2022–2024. Male soccer players overall at U17 demonstrated an over representation of players born at the beginning of the selection year, but this did not continue into the U20 and Senior age groups, questioning the validity of any perceived advantages at younger ages. The existence of RAEs were shown to only exist for players participating at age group level, with no RAEs being found for players playing-up in an older chronological age group than the respective competition they were qualified for. Finally, a larger proportion of nations demonstrated unequal birthdate distributions in male, but not female samples, when combining all age groups. We encourage coaches, scouts, and federations to be aware of and acknowledge the potential prevalence of RAEs within their nations and cohorts, and to consider the limitations associated with chronological age group bandings during their TID and (de)selection processes that may lead to (un)conscious age discrimination biases. Further, future research is warranted, which may aim to collaborate with federations and tournament organisers to formulate and explore new and existing strategies aimed at reducing RAEs in youth soccer.

## Supporting information

S1 Data Data. (XLSX)
